# Precise and long-term tracking of mitochondria in neurons using a bioconjugatable and photostable AIE luminogen†

**DOI:** 10.1039/d1sc06336g

**Published:** 2022-02-11

**Authors:** Hojeong Park, Guangle Niu, Chao Wu, Chungwon Park, Haixiang Liu, Hyokeun Park, Ryan T. K. Kwok, Jing Zhang, Benzhao He, Ben Zhong Tang

**Affiliations:** Department of Chemistry, Institute for Advanced Study, State Key Laboratory of Neuroscience and Division of Life Science, The Hong Kong University of Science and Technology Clear Water Bay Kowloon Hong Kong China tangbenz@cuhk.edu.cn; Shenzhen Institute of Aggregate Science and Technology, School of Science and Engineering, The Chinese University of Hong Kong 2001 Longxiang Boulevard, Longgang District Shenzhen City Guangdong 518172 China; Division of Life Science, State Key Laboratory of Molecular Neuroscience, HKUST Clear Water Bay Kowloon Hong Kong China; Department of Physics, HKUST Clear Water Bay Kowloon Hong Kong China; State Key Laboratory of Molecular Neuroscience, HKUST Clear Water Bay Kowloon Hong Kong China; HKUST-Shenzhen Research Institute No. 9 Yuexing 1st RD, South Area, Hi-tech Park, Nanshan Shenzhen 518057 China; State Key Laboratory of Crystal Materials, Shandong University Jinan 250100 China; Center for Advanced Materials Research, Advanced Institute of Natural Sciences, Beijing Normal University at Zhuhai Zhuhai 519085 China hebenzhao@bnu.edu.cn; Center for Aggregation-Induced Emission, SCUT-HKUST Joint Research Institute, State Key Laboratory of Luminescent Materials and Devices, South China University of Technology Guangzhou 510640 China

## Abstract

Tracking mitochondrial movement in neurons is an attractive but challenging research field as dysregulation of mitochondrial motion is associated with multiple neurological diseases. To realize accurate and long-term tracking of mitochondria in neurons, we elaborately designed a novel aggregation-induced emission (AIE)-active luminogen, TPAP-C5-yne, where we selected a cationic pyridinium moiety to target mitochondria and employed an activated alkyne terminus to achieve long-term tracking through bioconjugation with amines on mitochondria. For the first time, we successfully achieved the accurate analysis of the motion of a single mitochondrion in live primary hippocampal neurons and the long-term tracking of mitochondria for up to a week in live neurons. Therefore, this new AIEgen can be used as a potential tool to study the transport of mitochondria in live neurons.

## Abstract 2

A novel bioconjugatable and photostable AIE luminogen has been rationally synthesized for precise and long-term tracking of neuron mitochondria.

## Graphical abstract

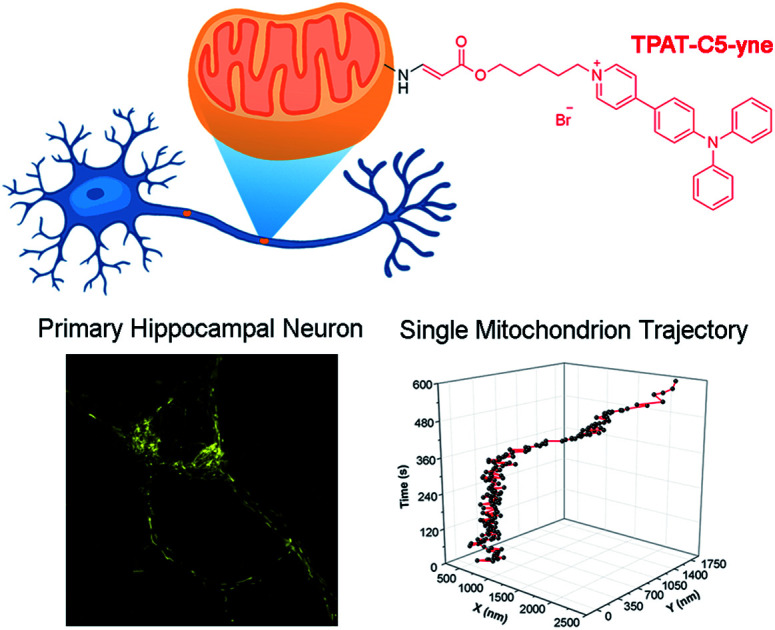

## Introduction

Mitochondria play a vital role in cells as organelles involving ATP synthesis by oxidative phosphorylation (OXPHOS), generation of free radical species (ROS), and apoptotic cell death.^1,2^ In neurons, mitochondria are exceptionally important for fulfilling the extraordinarily high metabolic rate of the central nervous system. Furthermore, most neuronal ATP is generated through OXPHOS, making neurons highly dependent on mitochondria.^3^ Due to the low regenerative potential of neurons, aged or damaged mitochondria must be removed and replaced with healthy ones at axon terminals.^4,5^ Failure in mitochondrial transport to maintain its healthy pool can have detrimental effects on neurons, and this is known to be associated with severe neurogenerative diseases including Huntington's disease, Parkinson's disease, and Alzheimer's disease.^6–9^ Therefore, it is crucial to monitor the mobility of mitochondria in real-time at the subcellular level to understand their key roles in the disease progression.^10^

Fluorescence microscopy is an effective tool to visualize and track mitochondria with high sensitivity and good temporal and spatial resolutions.^11–14^ It is routinely used in conjunction with fluorescent proteins and chemical probes to label intracellular mitochondria.^12,15,16^ To express fluorescent proteins, it is necessary to deliver plasmids containing mitochondrial localization sequences with fluorescent proteins into cells.^17–21^ However, such genetic manipulation is tedious as neurons are hard to transfect.^22,23^ On top of such complications, fluorescent proteins do not outperform synthetic fluorescent dyes in terms of photostability, diversity of colors, and brightness.^24^ Therefore, the use of small-molecule fluorescent dyes is more advantageous as the staining procedure is less laborious, while the dyes provide more favorable photophysical properties.^25,26^

To date, several cationic fluorescent dyes have been developed for selective staining of cellular mitochondria,^27,28^ however, these dyes still suffer from poor photostability, aggregation-caused quenching effect, low signal-to-noise ratio, high cytotoxicity or “always-on” behaviors.^15,22,29^ Recently, some hemi-cyanine-based dyes were developed to track the mitochondrial transport in primary neurons and glial cells.^22,30^ Nevertheless, due to the long acquisition time of confocal microscopy, images were acquired every 20 to 30 s and the detailed trajectory of the movement of a single mitochondrion could not be obtained.^22,30^

As a special type of fluorescent dye, aggregation-induced emission luminogens (AIEgens) show splendid photostability, and their “turn-on” behaviors result in a high signal-to-noise ratio upon fluorescence imaging.^31–33^ Therefore, many AIEgens have been synthesized to target different organelles, including mitochondria in cancer cell lines.^34–37^ However, when reported mitochondrial targeting AIEgens were applied to primary neurons, they showed unspecific binding and even distribution throughout the cells.^38^ To the best of our knowledge, there has been no successful attempt on using mitochondria-targeting AIEgens to monitor the motion of mitochondria in primary neurons.

**Fig. 1:**
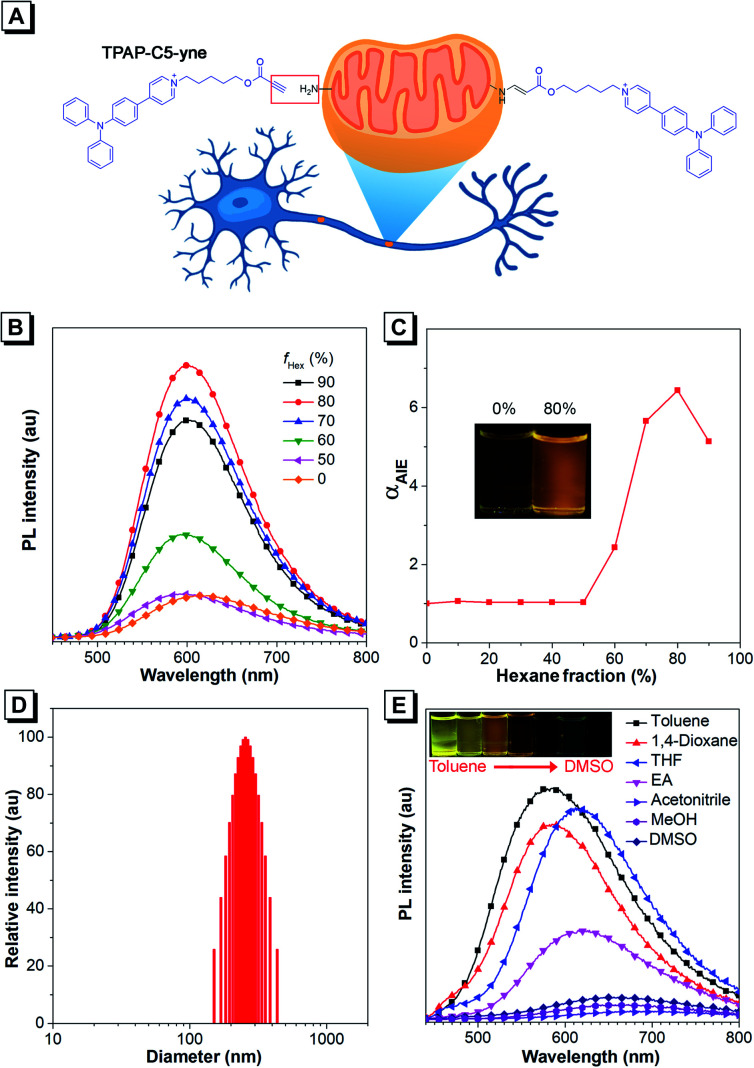
(A) Structure of TPAP-C5-yne (left) and its bioconjugate after reacting with an amine group (right). (B) PL spectra of TPAP-C5-yne (10 μM) in THF and THF/hexane mixtures with different hexane fractions (*f*_Hex_). (C) The plot of PL emission intensity *versus* the composition of the THF/hexane mixture containing TPAP-C5-yne. Inset: fluorescent photographs of TPAP-C5-yne in 0% and 80% hexane fractions under 365 nm UV irradiation. (D) Dynamic light scattering data of TPAP-C5-yne in THF/hexane mixtures containing 80% of hexane. (E) PL spectra of TPAP-C5-yne (10 μM) in different solvents.

Bioconjugation is a desirable strategy to achieve specific and stable binding between dye and mitochondria through covalent binding.^39–41^ In view of the outstanding advantages of AIEgens, we investigated whether AIEs could be bonded to mitochondria *via* the bioconjugation strategy and further realized the visual monitoring of mitochondrial mobility. Therefore, we elaborately introduced an activated alkyne unit into the AIE-active framework TPAP to achieve covalent binding with amines on mitochondria through bioconjugation considering the efficient reaction ability between activated alkyne and amines.^42^ Meanwhile, we selected a cationic pyridinium moiety to achieve the targeting of mitochondria.^12,43–45^ The resulting AIEgen, TPAP-C5-yne (Fig. 1A), successfully achieved the precise and up to 7 days tracking of mitochondria and the visualization of the motion of a single mitochondrion in live primary rat hippocampal neurons, which has not been reported to date.

## Results and discussion

### Design strategy, synthesis and photophysical properties

Regarding the molecular design, we intended to utilize the well-documented bioconjugation strategy to realize stable and long-term tracking of mitochondria.^39–41^ In the reported approaches of bioconjugation, azide–alkyne Huisgen cycloaddition is often used, but the toxicity of the copper catalyst limits its use in the biological system.^39,40^ Actually, metal-free “click” bioconjugation reactions have been developed in the past few years. Among them, we focused on highly efficient bond formation between amines and the electron-deficient ynone group.^41,42^ The Ynone group or so-called activated alkyne can form a stable connection with biomolecules containing amine groups. After rapid bioconjugation reaction, we believed that ynone group-containing AIEgens could be well retained in mitochondria, resulting in long-term tracking. Additionally, cationic pyridinium and triphenylphosphonium moieties have been reported to be capable of targeting mitochondria.^15,43–50^ Therefore, in the target molecule, we introduced an ynone group to achieve covalent binding with amines on mitochondria and selected the cationic pyridinium moiety to achieve the targeting of mitochondria. According to our knowledge, such a unique AIE probe that combines both mitochondrial targeting and *in situ* nontoxic, high-efficiency bioconjugation functions has not been reported to date. Then, we synthesized this target compound, TPAP-C5-yne and the final product was characterized by ^1^H NMR, ^13^C NMR, and HRMS spectroscopy to confirm its structure (Scheme S1 and Fig. S1–S9†). Detailed synthetic procedures are provided in the ESI.†

Firstly, we studied the photophysical properties of TPAP-C5-yne (Fig. 1B–E, S10 and S11†). TPAP-C5-yne exhibits good solubility and is weakly emissive in THF (Fig. 1B). However, when a poor solvent of hexane was added to its THF solution, its photoluminescence (PL) intensity gradually increased and reached a maximum at *f*_Hex_ = 80% (*Φ* = 34.7%, peak emission = 600 nm) due to restriction of intramolecular motion (RIM) in nanoaggregates (Fig. 1B and C). The particle size analysis further reveals the existence of nanoaggregates with an average size of 256 nm in THF/hexane mixtures containing 80% hexane, indicating that TPAP-C5-yne is a typical AIE-active molecule (Fig. 1D).

Next, the solvatochromic behavior of TPAP-C5-yne was investigated. Although TPAP-C5-yne shows a strong yellow emission in low-polarity toluene, as the solvent polarity increases, the intensity becomes weak and red-shifts due to the intramolecular charge transfer (ICT) effect (Fig. 1E). This suggests that TPAP-C5-yne can serve as a “light-up” probe as it interacts with the low polarity and hydrophobic sites of protein and other biomolecules.^35^

### Bioconjugation between the activated alkyne and amine group

**Fig. 2:**
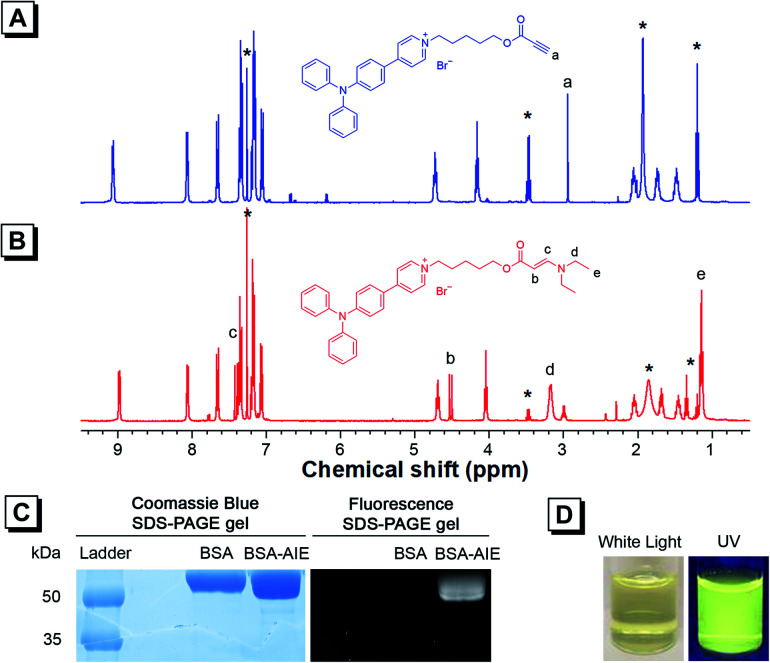
^1^H NMR spectra of (A) TPAP-C5-yne and (B) TPAP-C5-aa in CDCl_3_. The solvent peaks are marked with asterisks. (C) SDS-PAGE gels showing protein bands of BSA and BSA-TPAP-C5-yne on coomassie blue (left) and fluorescence (right). (D) Photographs of BSA-TPAP-C5-yne aqueous dispersion under white light (left) and UV irradiation (right).

As TPAP-C5-yne comprises an activated alkyne, we confirmed its ability to react with the amine group by adding *N*,*N*-diethylamine into TPAP-C5-yne. ^1^H NMR, ^13^C NMR, and HRMS spectra of TPAP-C5-aa were obtained (Fig. S12–S14†). In the ^1^H NMR spectra, the ethynyl proton of TPAP-C5-yne resonates at *δ* 2.94 (Fig. 2A), which is absent after reacting with the secondary amine (Fig. 2B). Meanwhile, three new peaks resonate at *δ* 7.43, 4.53, and 3.18, which are assignable to the resonances of the HC=CH group and CH_2_ group next to the N atom, respectively (Fig. 2B). Moreover, in the ^13^C NMR spectra, the peaks at *δ* 75.29 and 74.73 corresponding to the ethynyl carbons of TPAP-C5-yne are absent in the ^13^C NMR spectra of TPAP-C5-aa. Meanwhile, two new peaks associated with the resonances of vinyl carbons appeared at *δ* 155.22 and 83.14 in the spectra of TPAP-C5-aa (Fig. S15†). These results further confirmed the successful click reaction between the activated alkyne and amine group.

After verifying that TPAP-C5-yne successfully reacted with the amine group, we examined the formation of the BSA-TPAP-C5-yne conjugate by sodium dodecyl sulfate polyacrylamide gel electrophoresis (SDS-PAGE) (Fig. 2C). The BSA-TPAP-C5-yne conjugate exhibits yellow emission that is consistent with that of TPAP-C5-yne in low-polarity toluene, suggesting that TPAP-C5-yne may interact with the hydrophobic sites of the protein (Fig. 2D). NMR and SDS-PAGE results further conclude that TPAP-C5-yne can be used to functionalize proteins in an aqueous solution without catalysts.

Intracellular ROS cause a wide range of damage through the oxidation of biomolecules, and the imbalance of ROS and antioxidants results in oxidative stress.^51,52^ As numerous AIEgens with a donor–acceptor dyad molecular skeleton previously developed can generate ROS, they are highly phototoxic.^51^ Neurons are extremely susceptible to oxidative stress because of their high metabolic rate and low regenerative capacity.^51^ Therefore, it is crucial to assess the ROS generation potential of TPAP-C5-yne for its application in neuronal cell imaging. Accordingly, we tested the ^1^O_2_ generation ability of TPAP-C5-yne using 9,10-anthracenediyl-bis(methylene)dimalonic acid (Fig. S16†), and the results showed that the ^1^O_2_ generation of TPAP-C5-yne was minimal.

### Cytotoxicity, photostability and Co-localization

**Fig. 3:**
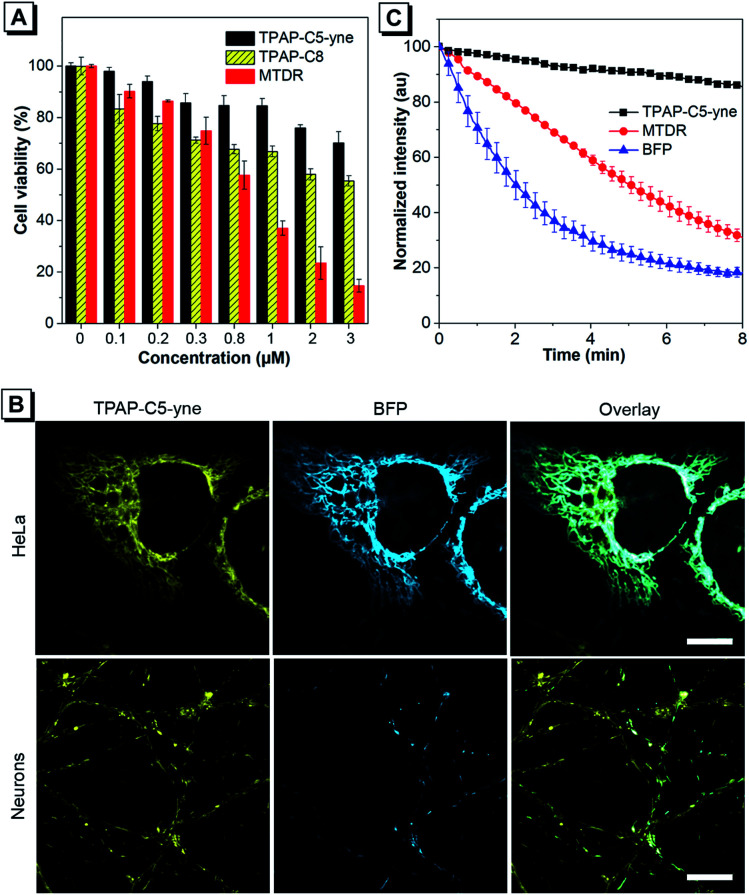
(A) Cell viability of neurons stained with TPAP-C5-yne, TPAP-C8 and MTDR. (B) Co-localized images of HeLa cells and neurons stained and transfected with TPAP-C5-yne (300 nM) and Mito-BFP respectively. Scale bar: 10 μm. (C) Photostability of TPAP-C5-yne, MTDR, and BFP under continuous irradiation.

The cytotoxicity of TPAP-C5-yne was further evaluated using the 3-(4,5-dimethyl-2-thiazolyl)-diphenyltetrazolium bromide (MTT) assay in HeLa cells and primary rat hippocampal neurons (Fig. 3A and S17†). We synthesized TPAP-C8 without an activated alkyne as a control to test the effect of activated alkyne on cytotoxicity (Scheme S2 and Fig. S18–S20†) and utilized the commercially available MitoTracker Deep Red (MTDR) as another control. TPAP-C5-yne shows negligible cytotoxicity in both HeLa cells and neurons. In contrast, TPAP-C8 is slightly more cytotoxic to neurons compared to HeLa cells, whereas MTDR is cytotoxic to both cells.

Subsequently, live-cell imaging in HeLa cells and neurons was performed. TPAP-C5-yne stains specifically the mitochondria of HeLa cells and neurons (Fig. S21–S23†). TPAP-C8 stains mitochondria in both HeLa cells and neurons (Fig. S24†) but induces a morphological change of the cells. Then, the co-staining experiment with Mito-BFP was performed to avoid any possible dye–dye interactions inside the cell. The co-staining image of HeLa cells with TPAP-C5-yne and Mito-BFP shows good localization with a Pearson coefficient value of 0.9355, however, only partial overlapping was observed in neurons due to the low transfection efficiency of neurons (Fig. 3B).^53^ Photostability of fluorescent materials is a critically important factor to achieve long-term imaging. TPAP-C5-yne shows negligible change over 8 min, while MTDR and Mito-BFP are bleached rapidly under continuous laser irradiation (Fig. 3C and S25†).

### Mitochondrial movement tracking and long-term staining

**Fig. 4:**
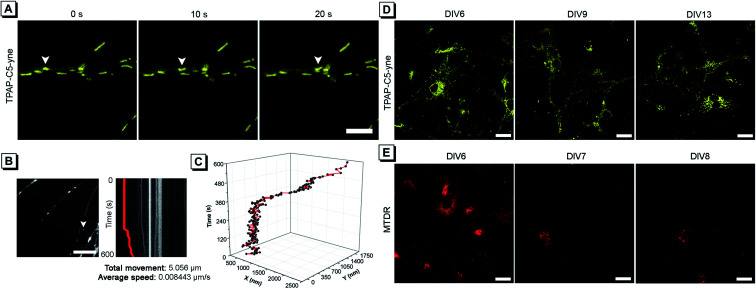
(A) Confocal microscopy images of neurons stained with TPAP-C5-yne. White arrows indicate a single mitochondrion. Scale bar: 5 μm. (B) Screenshot of a real-time image acquired using an EM-CCD camera. White arrow indicates a mitochondrion and a kymograph generated from the indicated mitochondrion. Scale bar: 10 μm. (C) Track of the movement of a single mitochondrion is presented in the 3D diagram. (D and E) Confocal microscopy images of neurons stained with (D) TPAP-C5-yne and (E) MTDR on DIV 6. Scale bar: 20 μm.

Considering the low cytotoxicity and mitochondrial targeting of TPAP-C5-yne and the confirmation of rapid bioconjugation reaction with amine group-containing biomolecules in mitochondria, we then tested its capability of precise mitochondrial imaging. Under a confocal fluorescence microscope, we observed an apparent change in the mitochondrial location. To track the movement, fluorescence images were acquired every 10 s and the moving mitochondrion is indicated with a white arrow (Fig. 4A and ESI Video†). To further investigate the movement of a single mitochondrion in neurons more precisely, we acquired real-time images using an iXon Ultra electron-multiplying CCD (EM-CCD) camera every 2 s, and a kymograph was generated for the mitochondrion, indicated with a white arrow (Fig. 4B). Here, it should be noted that some hemi-cyanine-based dyes were also reported that could realize the tracking of mitochondrial transport in primary neurons and glial cells recently.^22,30^ However, images were acquired every 20 to 30 s and the detailed trajectory of a single mitochondrion movement could not be obtained due to the long acquisition time of confocal microscopy.^54^ In the present system, we could achieve precise imaging of a single mitochondrion. According to the imaging analysis, this mitochondrion has a total movement (net displacement) of 5.056 μm and an average speed (total net displacement/total time) of 0.008443 μm s^−1^ for 10 min (Fig. 4B). The position of a mitochondrion in the *x* and *y* axis *vs*. time was plotted in a 3D diagram (Fig. 4C).

Considering the superior photostability and imaging ability in the mitochondria of TPAP-C5-yne, we then explored its possibility to be applied for long-term imaging of neurons. Neurons were stained on day *in vitro* (DIV) 6 with TPAP-C5-yne and MTDR, respectively. Neurons stained with TPAP-C5-yne did not show any apparent morphology change. Images could be acquired for a week with minor changes in the location of TPAP-C5-yne (Fig. 4D). In contrast, the neurons stained with MTDR underwent severe changes in morphology within a day (Fig. 4E). Results as shown in Fig. 4 help us to confirm that TPAP-C5-yne is photostable and biocompatible to be used for studying the dynamic movement of mitochondria accurately over a long period of time. Therefore, this work opens up new possibilities of developing new AIEgens for neuroscience applications through structural modifications.

## Conclusions

In summary, we synthesized a polarity-sensitive and bioconjugatable AIE probe TPAP-C5-yne. It can specifically label mitochondria in live cancer cells and primary neurons at a low concentration. We successfully tracked the precise motion of a single mitochondrion in neurons for the first time using TPAP-C5-yne. Because of its superior photostability and rapid bioconjugation reaction with amine group containing biomolecules in mitochondria, TPAP-C5-yne can be used to achieve long-term tracking of mitochondria in neurons with minor perturbations for a week, and outperforms the highly cytotoxic commercially available dye. This work opens new possibilities in developing new fluorescent probes for neuroscience applications.

## Author contributions

H. P., B. H., and B. Z. T. conceived and designed the experiments. B. H. and H. P. performed the synthesis. H. P., B. H., C. W. and C. P. performed the fluorescent imaging experiment. G. N., H. P., J. Z and R. T. K. K. took part in the discussion and gave important suggestions. H. P., B. H. and B. Z. T. wrote the paper with comments from all authors.

## Conflicts of interest

The authors declare no conflicts of interest.

## Supplementary Material

SC-013-D1SC06336G-s001

SC-013-D1SC06336G-s002
