# Supplementary material for: Precise and long-term tracking of mitochondria in neurons using a bioconjugatable and photostable AIE luminogen
Source: Chem Sci. 2022 Feb 11;13(10):2965–70. doi: 10.1039/d1sc06336g (PMC8905947; doi:10.1039/d1sc06336g)
Supplement: SC-013-D1SC06336G-s001 [file SC-013-D1SC06336G-s001.pdf]

Electronic Supplementary Material (ESI) for Chemical Science.
This journal is © The Royal Society of Chemistry 2022

Electronic Supplementary Information

Precise and Long-Term Tracking of Mitochondria in Neurons Using a Bioconjugatable and Photostable AIE Luminogen

Hojeong Park,[a] Guangle Niu,[g] Chao Wu,[c] Chungwon Park,[c] Haixiang Liu,[f] Hyokeun Park,[c,d,e] Ryan T. K. Kwok,[a,f] Jing Zhang,[a] Benzhao He,[,a,h] and Ben Zhong Tang[*,a,b,f,i]*

[a]*Department of Chemistry, Institute for Advanced Study, State Key Laboratory of Neuroscience and Division of Life Science, The Hong Kong University of Science and Technology, Clear Water Bay, Kowloon, Hong Kong, China. E-mail: tangbenz@cuhk.edu.cn*

[b]*Shenzhen Institute of Aggregate Science and Technology, School of Science and Engineering, The Chinese University of Hong Kong, Shenzhen, 2001 Longxiang Boulevard, Longgang District, Shenzhen City, Guangdong 518172, China*

[c]*Division of Life Science, State Key Laboratory of Molecular Neuroscience, HKUST, Clear Water Bay, Kowloon, Hong Kong, China*

[d]*Department of Physics, HKUST, Clear Water Bay, Kowloon, Hong Kong, China*

[e]*State Key Laboratory of Molecular Neuroscience, HKUST, Clear Water Bay, Kowloon, Hong Kong, China*

[f]*HKUST-Shenzhen Research Institute, No. 9 Yuexing 1st RD, South Area, Hi-tech Park, Nanshan, Shenzhen 518057, China*

[g]*State Key Laboratory of Crystal Materials, Shandong University, Jinan 250100, China*

[h]*Center for Advanced Materials Research, Advanced Institute of Natural Sciences, Beijing Normal University at Zhuhai, Zhuhai 519085 China. Email: hebenzhao@bnu.edu.cn*

[i]*Center for Aggregation-Induced Emission, SCUT-HKUST Joint Research Institute, State Key Laboratory of Luminescent Materials and Devices, South China University of Technology, Guangzhou 510640, China*

Materials and Methods

General Information

All chemicals were purchased from J&K Chemical Co. and Sigma-Aldrich and used as received without further purification. Anhydrous tetrahydrofuran (THF) and toluene were distilled from sodium benzophenone ketyl under dry nitrogen right before fluorescence property investigation. Deionized water was used throughout this study. All aqueous solutions were freshly prepared with deionized water. ^{1}H NMR (400 MHz) and ^{13}C NMR (100 MHz) spectra were recorded on a Bruker ARX 400 spectrometer using tetramethylsilane (TMS) as internal standard. High-resolution mass spectra (HRMS) were recorded on a GCT premier CAB048 mass spectrometer operated in a MALDI-TOF mode. Absorption spectra were measured on a PerkinElmer Lambda 365 UV/Vis spectrophotometer. Steady-state photoluminescence (PL) spectra were measured on a Perkin-Elmer spectrofluorometer LS55. Absolute fluorescence quantum yield was measured by a Hamamatsu Quantaurus-QY Plus UV-NIR Absolute PL quantum yield spectrometer. Hydrated diameter of aggregates was measured at room temperature by NanoBrook 90 Plus Zeta Particle Size Analyzer.

Sodium Dedecyl Sulfate Polyacrylamide Gel Electrophoresis (SDS-PAGE)

0.5 mg/mL bovine serum albumin (BSA) stock solution was obtained by diluting in phosphate-buffered saline (PBS, pH 7.4). 10 µl of 1 mM TPAP-C5-yne prepared in anhydrous dimethyl sulfoxide (DMSO) was added to 1 mL of BSA stock solution and reacted for 30 min at the room temperature. 5 µg of protein was loaded to each well of 12 % polyacrylamide gel. Fluorescence and Coomassie blue SDS-PAGE gels were visualized using Bio-rad Laboratories ChemiDoc Touch Imaging System.

$^1O_2$ Generation Detection

9-10-Anthracenediyl-bis(methylene) dimalonic acid (ABDA) solution (10 mM) was prepared in DMSO and added to 2 mL of TPAT-C5-yne or Rose Bengal suspension (10 µM) in PBS. Absorption spectra of ABDA between 340 to 420 nm were measured at various white irradiation (4.76 mW cm^{-2}) time.

Cell Cultures and Live Cell Imaging

HeLa and Primary Neuronal Cultures

HeLa cells were cultured and regularly passaged in the culture medium (Dulbecco's modified Eagle medium (DMEM), supplemented with 10 % fetal bovine serum (FBS) and 1% penicillin and streptomycin) at 5 % CO_2/air at 37 °C in a humidified incubator. A day prior to the experiment, the cells were split, and approximately 8 × 10^5 cells were seeded onto each coverslip in 35 mm petri dish. Hippocampal neurons were isolated from postnatal day 0 (P0) Sprague Dawley rat pups obtained from the Animal and Plant Care Facility at the Hong Kong University of Science and Technology (HKUST) and cultured using a similar protocol as previously described.[1] The hippocampal tissues were dissected and digested briefly with papain containing DNase I. After digestion, the density of dissociated neurons was determined. About 5 × 10^4 cells were plated onto 35 mm clear cover glass-bottom confocal petri dish coated with poly-*D*-lysine. Neurobasal medium supplemented with 2 % B-27 supplement, 0.5 mM Glutamax supplement, 10 % fetal bovine serum (FBS) and 1% penicillin and streptomycin), was used as a culture medium. After 50 h, 20 µM 5-fluoro-2′-deoxyuridine was added to inhibit the proliferation of glial cells. The neurons were incubated at 5% CO_2/air at 37 °C in a humidified incubator and imaging

experiments were performed at day *in vitro* (DIV) 6-21. These experiments were performed in compliance with the relevant laws and intuitional guidelines. The institutional committee had approved the experiment.

Transfection of HeLa and Primary Neuronal Cultures

Mito-BFP (a gift from G. Voeltz, Addgene plasmid 49151) was extracted from DH5*α Escherichia coli* with PureLink® HiPure Plasmid Maxiprep Kit (Thermo Fisher Scientific). Transfection of plasmid encoding Mito-BFP was performed using lipofectamine 2000 (Thermo Fisher Scientific) according to the manufacturer's instructions in Opti-MEM Reduced-Serum Medium (Thermo Fisher Scientific). For primary neuronal cultures, transfection reagent was added at DIV 7 and incubated for 4 h prior to changing to fresh culture medium. Confocal fluorescence imaging was performed 24 to 48 h after transfection.

Live Cell Imaging

HeLa and hippocampal neuron cells were incubated in Hank's balanced salt solution (HBSS, pH 7.4) containing 300 nM of TPAP-C5-yne, TPAP-C8 or MitoTracker Deep Red FM (MTDR) (300 μM TPAP-C5-yne and MTDR stock solutions were prepared in DMSO) at 37 ℃ in 5% CO_2 for 20 min before live cell imaging. For co-stain imaging, HeLa and hippocampal neuron cells transfected with Mito-BFP plasmid, were incubated with 300 nM of TPAP-C5-yne at 37 ℃ in 5% CO_2 for 30 min before live cell imaging. After incubation, the cells were washed three times with HBSS. Confocal fluorescence imaging was obtained on Zeiss LSM 800 confocal scanning microscope (excitation = 405 nm and emission collection: 410-500 nm for BFP and 500-650 nm for TPAP-C5-yne). For the photostability test, the cells either containing different dyes or expressing BFP were continuously irradiated with confocal laser for 8 min (excitation = 405 nm for TPAP-C5-yne and BFP, and 640 nm for MTDR, laser power = 4%).

Real-time two-dimensional live cell images were obtained at the room temperature at 0.5 Hz for 600 s using a Olymus IX-73 objective inverted fluorescence microscope, equipped with a 100X oil immersion objective (NA =1.49, UAPON 100xOTIRF, Olymus). A 405 nm laser (CrystaLaser, Reno, NV) was used to excite TPAP-C5-yne. ZT488/532rpc-UF1 (Chroma) dichroic mirror and a ET580/60M (Chroma) emission filter were placed inside the microscope filter cube to collect the fluorescence signals. The emission signals were imaged onto a iXon-897 Andor electron-multiplying CCD (EM-CCD). Kymographs were generated from 10-time-time-laspe movie and analyzed using the ImageJ macro Kymolyzer.[2] Single-particle tracking trajectories for images were obtained using custom-written IDL coded programs.[3] Throughout the imaging experiments, the cells were perfused with 4K Tyrode's solution (150 mM NaCl, 4 mM KCl, 2 mM $MgCl_2$, 2 mM CaCl, 10 mM HEPES and 10 mM glucose, pH 7.2).

Cell Viability Test

The cytotoxicity of TPAP-C5-yne, TPAP-C8 and MTDR were investigated by the standard tetrazolium salt (3-(4,5-dimethylthiazol-2-yl)-2,5-diphenyltetrazolium bromide(MTT) assay using a similar protocol as previously described.[4] 5×10^3 cells were seeded onto each well of 96-well plate and allowed to adhere for 24 h for HeLa cells and 6 days for hippocampal neuron cells. 200 μL of culture medium containing one of: TPAP-C5-yne, TPAP-C8 or MTDR was added into each well (concentration range: 0.1 ~ 3 μM) and culture medium with only DMSO was added into wells to serve as a negative control group. The cells were incubated for 24 h at 5 % CO_2/air at 37 °C in a humidified incubator. MTT solution (5 mg/mL in culture medium) was added into each well and cells were incubated for another 4 h. The MTT solution was replaced with 100 μL of DMSO for each well to lysis

cells and dissolve water insoluble formazan. 96-well plate was shaken on the shaker for 3 min and the optical density (OD) readings at 570 nm were measured using a plate reader.

Synthetic Details

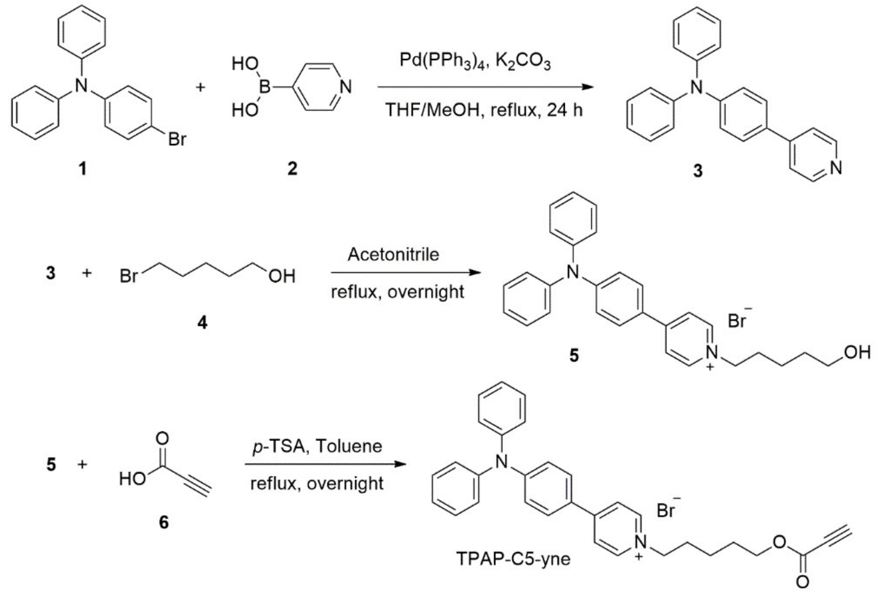

Scheme S1. Synthetic route to TPAP-C5-yne.

Synthesis of compound 3. In a two-neck round-bottomed flask, a solution of compound **1** (3.24 g, 10 mmol), compound **2** (1.48 g, 12 mmol), $Pd(PPh_3)_4$ (578 mg, 0.5 mmol) and K_2CO_3 (5.53 g, 40 mmol) were refluxed under nitrogen in THF and MeOH (1:1, *v*/*v*, 100 mL) at 80 ^oC for 24 h. After cooling to room temperature, the solvent was removed under reduced pressure and the residue was extracted with dichloromethane (DCM) and water. The crude product was purified by a silica gel chromatography using hexane/DCM (3:1 v/v) as the eluent to yield compound **3** as a white solid (2.90 g, 90% yield). ^1H NMR (400 MHz, $CDCl_3$) δ (ppm): 8.62 (d, J = 6.4 Hz, 2H), 7.53 (d, J = 8.4 Hz, 2H), 7.48 (d, J = 6.4 Hz, 2H), 7.29 (t, J = 7.6 Hz, 4H), 7.16-7.13 (m, 6H), 7.08 (t, J = 7.2 Hz, 2H). ^{13}C NMR (100 MHz, $CDCl_3$) δ (ppm): 150.33, 149.05, 147.73, 147.36, 131.06, 129.56, 127.76, 125.10, 123.72, 123.03, 120.98. HRMS (MALDI-TOF): *m/z* calcd for $[C_{23}H_{20}N_2]^+$ 323.1548 ($[M+H]^+$), found 323.1541.

Synthesis of compound 5. In a two-neck round-bottomed flask, compound **3** (966 mg, 3 mmol) and compound **4** (1.00 g, 6 mmol) were refluxed at 80 ^oC overnight in acetonitrile (50 mL) in a two-neck round-bottomed flask. After cooling to room temperature, the mixture was added into 150 mL of ice-cold diethyl ether with vigorous stirring. The precipitates were separated by centrifugation and washed three times with diethyl ether and dried in vacuum at 40 ^oC to a constant weight. Eventually, compound 5 was obtained as a yellow powder (1.24 g, 85% yield). ^1H NMR (400 MHz, $CDCl_3$) δ (ppm): 9.30 (d, J = 6.8 Hz, 2H), 8.09 (d, J = 6.8 Hz, 2H), 7.67 (d, J = 8.8 Hz, 2H), 7.34 (t, J = 7.6 Hz, 4H), 7.19-7.14 (m, 6H), 7.06 (d, J = 9.2 Hz, 2H), 4.87 (t, J = 7.6 Hz, 2H), 3.39 (m, 2H), 2.12-2.03 (m, 2H), 1.96-1.87 (m, 2H), 1.61-1.51 (m, 2H). ^{13}C NMR (100 MHz, $CDCl_3$) δ (ppm): 155.12, 152.35, 145.91, 144.39, 129.95, 129.16, 126.40, 125.51, 124.05, 122.86, 120.42, 62.62, 60.14, 33.54, 31.89, 30.86. HRMS (MALDI-TOF): *m/z* calcd for $[C_{28}H_{29}N_2O]^+$ 409.2274 ($[M]^+$), found 409.2281.

Synthesis of TPAP-C5-yne. In a 100 mL round-bottom flask equipped with a Dean–Stark apparatus were added compound **5** (488 mg, 1 mmol), propiolic acid **6** (210 mg, 3 mmol) and *p*-TSA (19 mg, 0.1 mmol) and 50 mL of dry toluene. The mixture was allowed to reflux overnight with constant removal of yielded water. After cooling to room temperature, the solvent was removed under reduced pressure, the residue was purified by silica gel chromatography using DCM/MeOH (from 95:5 to 85:15, *v/v*) as the eluent to yield compound TPAP-C5-yne as a yellow powder (270 mg, 50% yield). ^{1}H NMR (400 MHz, $CDCl_3$) δ (ppm): 9.07 (d, J = 6.8 Hz, 2H), 8.07 (d, J = 6.4 Hz, 2H), 7.67 (d, J = 9.2 Hz, 2H), 7.35 (t, J = 8.0 Hz, 4H), 7.20-7.15 (m, 6H), 7.06 (t, J = 9.2 Hz, 2H), 4.73 (t, J = 7.2 Hz, 2H), 4.17 (m, 2H), 2.94 (s, 1H), 2.10-2.01 (m, 2H), 1.78-1.71 (m, 2H), 1.52-1.44 (m, 2H). ^{13}C NMR (100 MHz, $CDCl_3$) δ (ppm): 155.21, 152.84, 152.34, 145.94, 144.29, 129.96, 129.19, 126.41, 125.51, 124.08, 122.97, 120.44, 75.29, 74.73, 65.81, 60.37, 31.17, 27.75, 22.59. HRMS (MALDI-TOF): *m/z* calcd for $[C_{31}H_{29}N_2O_2]^+$ 461.2224 ($[M]^+$), found 461.2194.

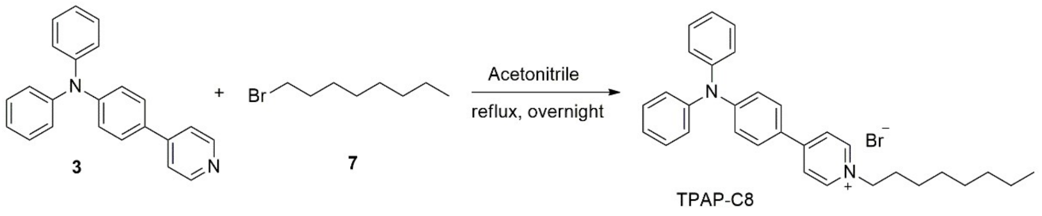

Scheme S2. Synthetic route of TPAP-C8.

Synthesis of TPAP-C8. In a two-neck round-bottomed flask, compound **3** (966 mg, 3 mmol) and compound **7** (1.16 g, 6 mmol) were refluxed at 80 °C overnight in acetonitrile (50 mL) in a two-neck round-bottomed flask. After cooling to room temperature, the mixture was added into 150 mL of ice-cold diethyl ether with vigorous stirring. The precipitates were separated by centrifugation and washed three times with diethyl ether and dried in vacuum at 40 °C to a constant weight. Eventually, TPAP-C8 was obtained as a yellow powder (1.36 g, 88% yield). ^{1}H NMR (400 MHz, $CDCl_3$) δ (ppm): 9.19 (d, J = 6.8 Hz, 2H), 8.09 (d, J = 6.8 Hz, 2H), 7.66 (d, J = 8.8 Hz, 2H), 7.35 (t, J = 8.0 Hz, 4H), 7.20-7.15 (m, 6H), 7.07 (d, J = 8.8 Hz, 2H), 4.80 (t, J = 7.6 Hz, 2H), 2.03-1.96 (m, 2H), 1.25-1.21 (m, 10H), 0.84 (t, J = 7.2 Hz, 3H). ^{13}C NMR (100 MHz, $CDCl_3$) δ (ppm): 155.08, 152.34, 145.93, 144.28, 129.97, 129.14, 126.41, 125.52, 124.06, 122.87, 120.43, 60.76, 31.87, 31.79, 29.17, 29.14, 26.25, 22.69, 14.18. HRMS (MALDI-TOF): *m/z* calcd for $[C_{31}H_{35}N_2]^+$ 435.2795 ($[M]^+$), found 435.2803.

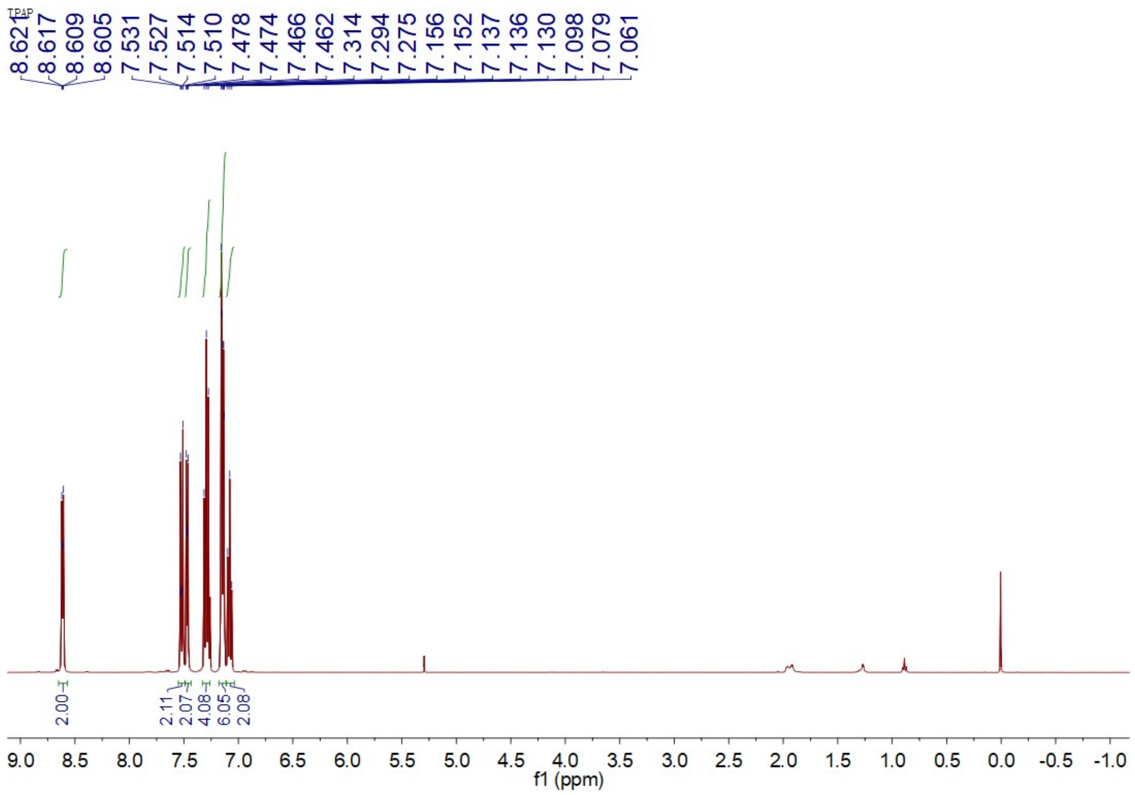

Figure S1. ^{1}H NMR (400 MHz, $CDCl_3$) spectrum of **3**.

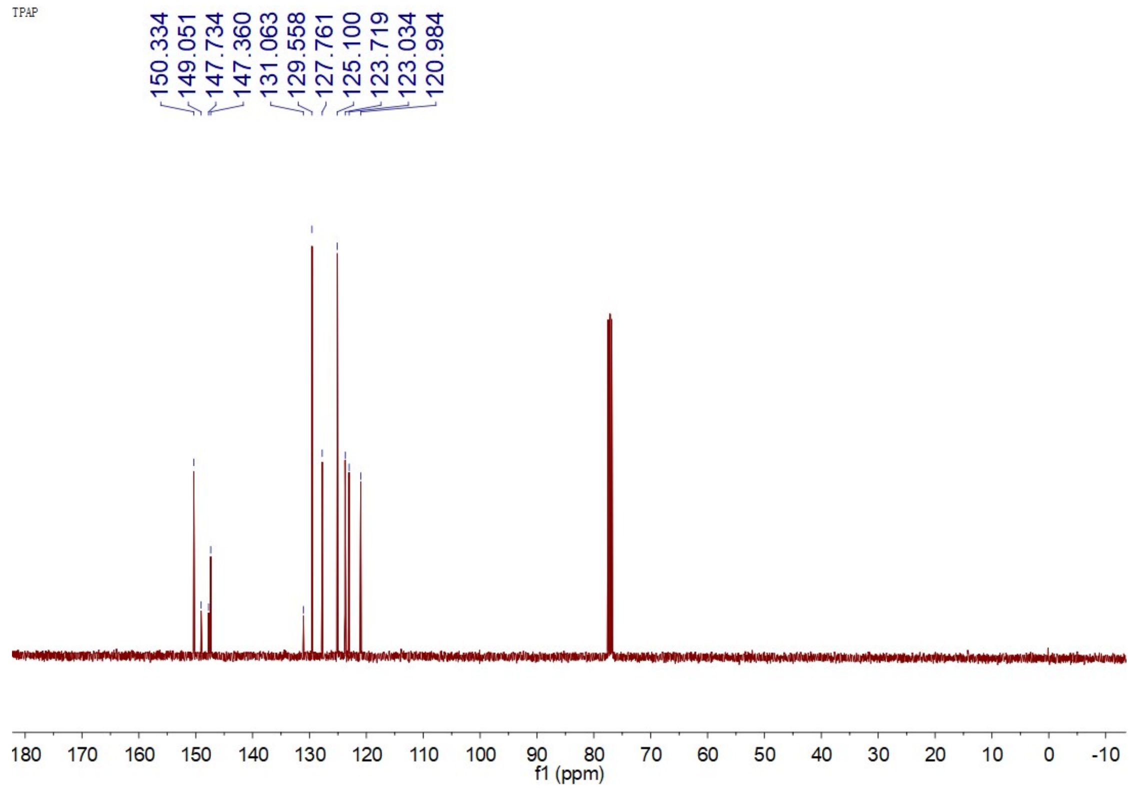

Figure S2. ^{13}C NMR (100 MHz, $CDCl_3$) spectrum of **3**.

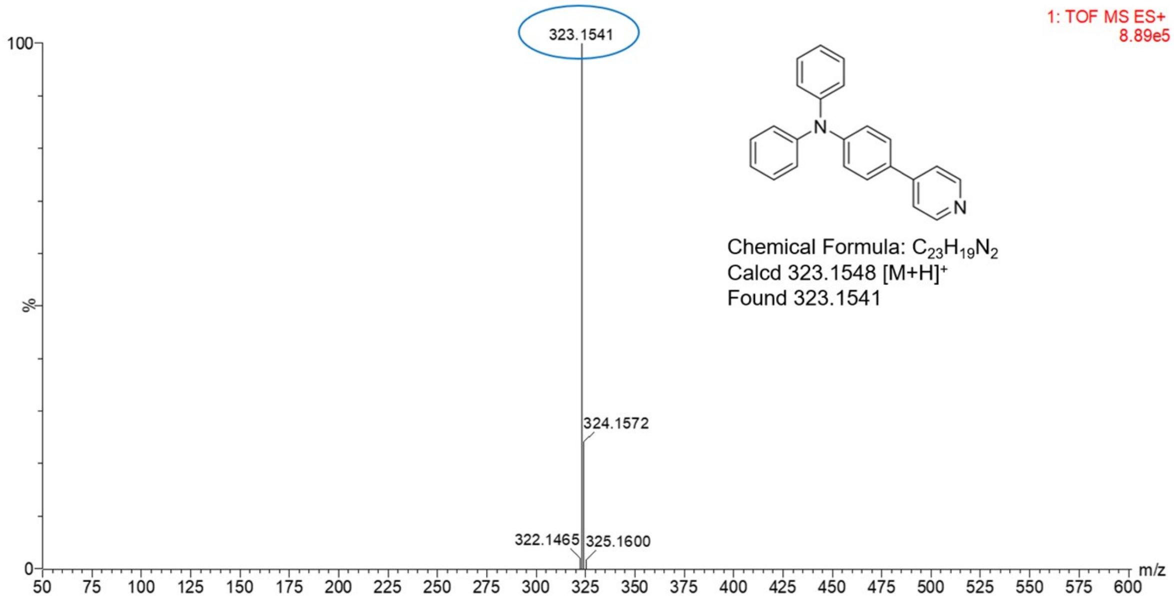

Figure S3. High-resolution mass spectrum of **3**.

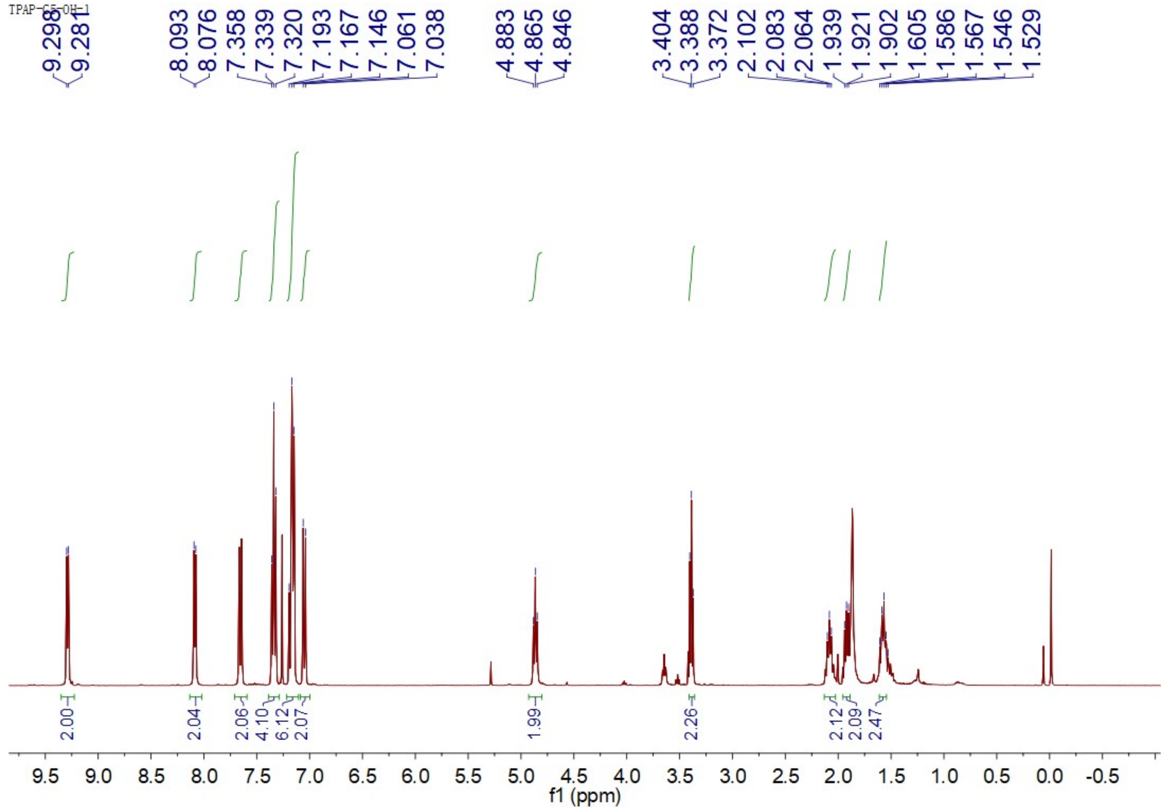

Figure S4. ^{1}H NMR (400 MHz, $CDCl_3$) spectrum of **5**.

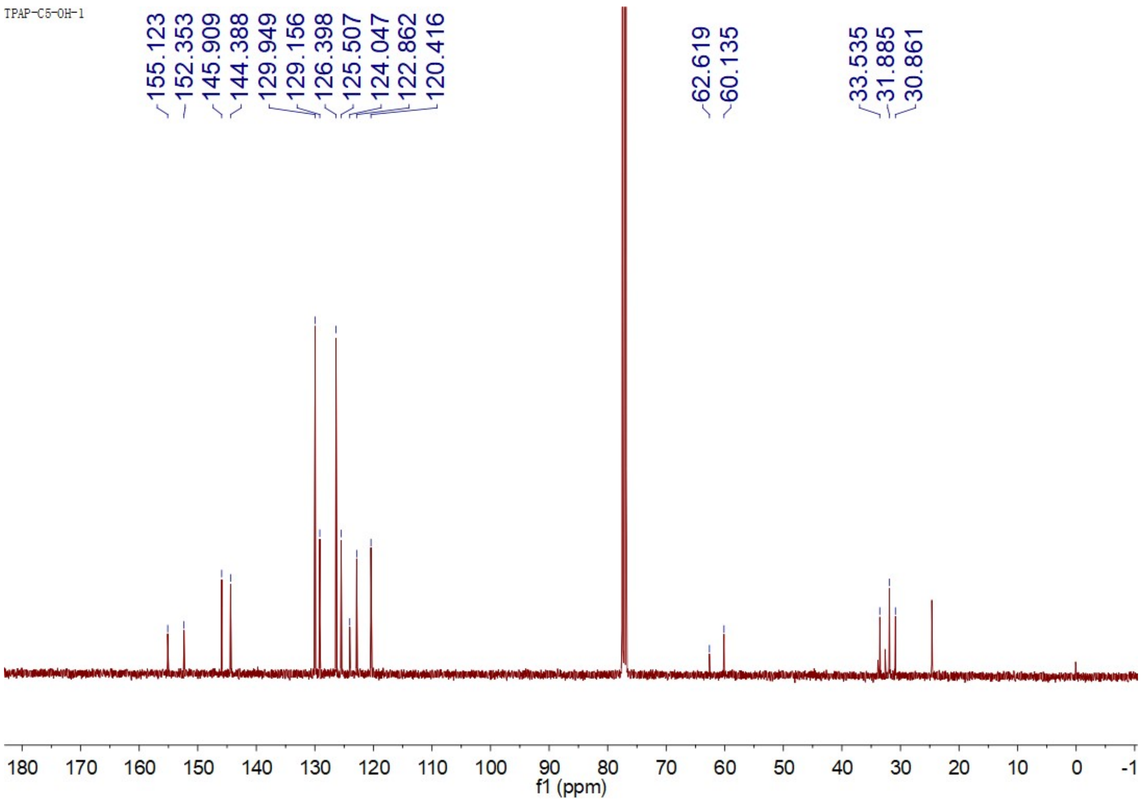

Figure S5. ^{13}C NMR (100 MHz, $CDCl_3$) spectrum of **5**.

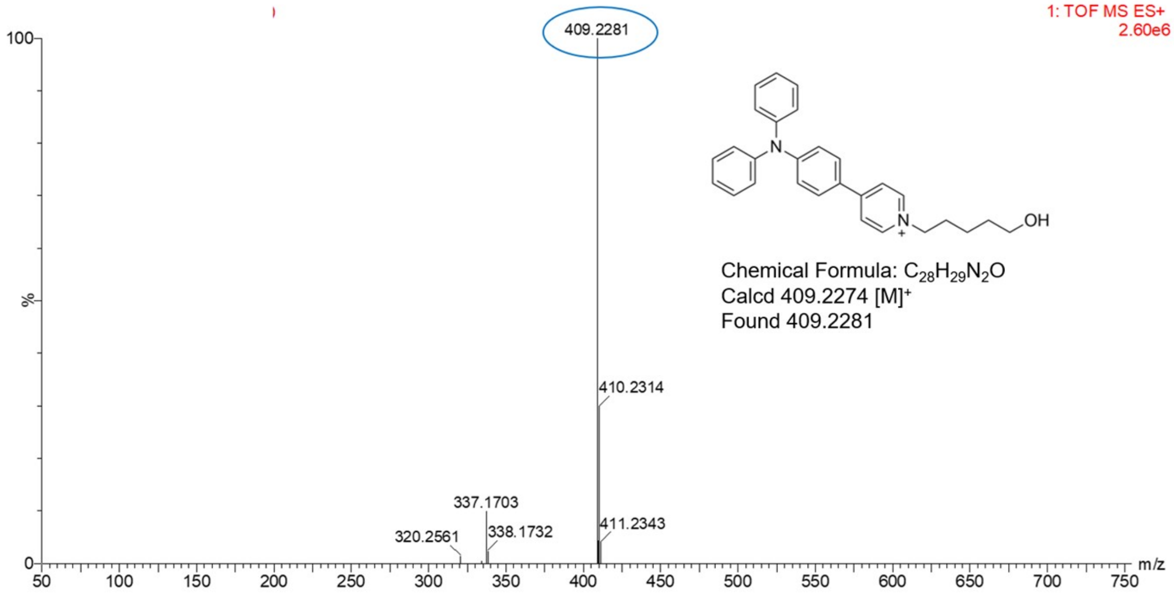

Figure S6. High-resolution mass spectrum of **5**.

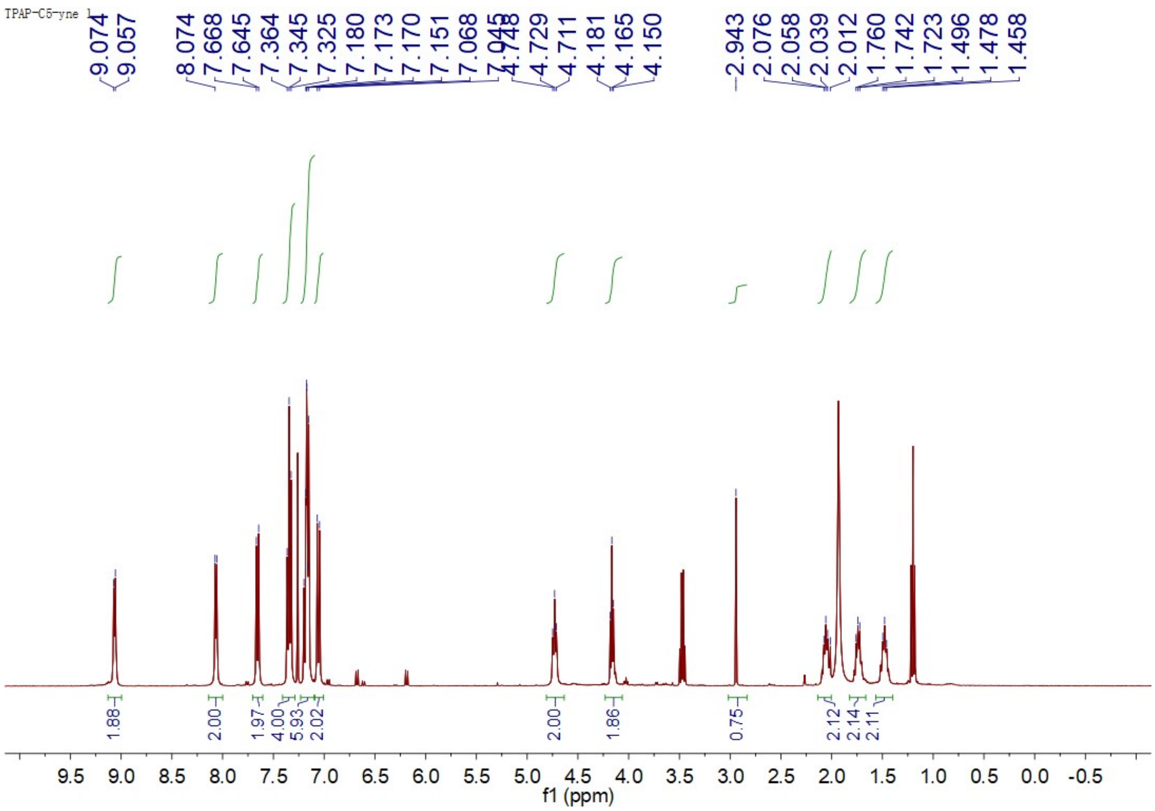

Figure S7. ^{1}H NMR (400 MHz, $CDCl_3$) spectrum of TPAP-C5-yne.

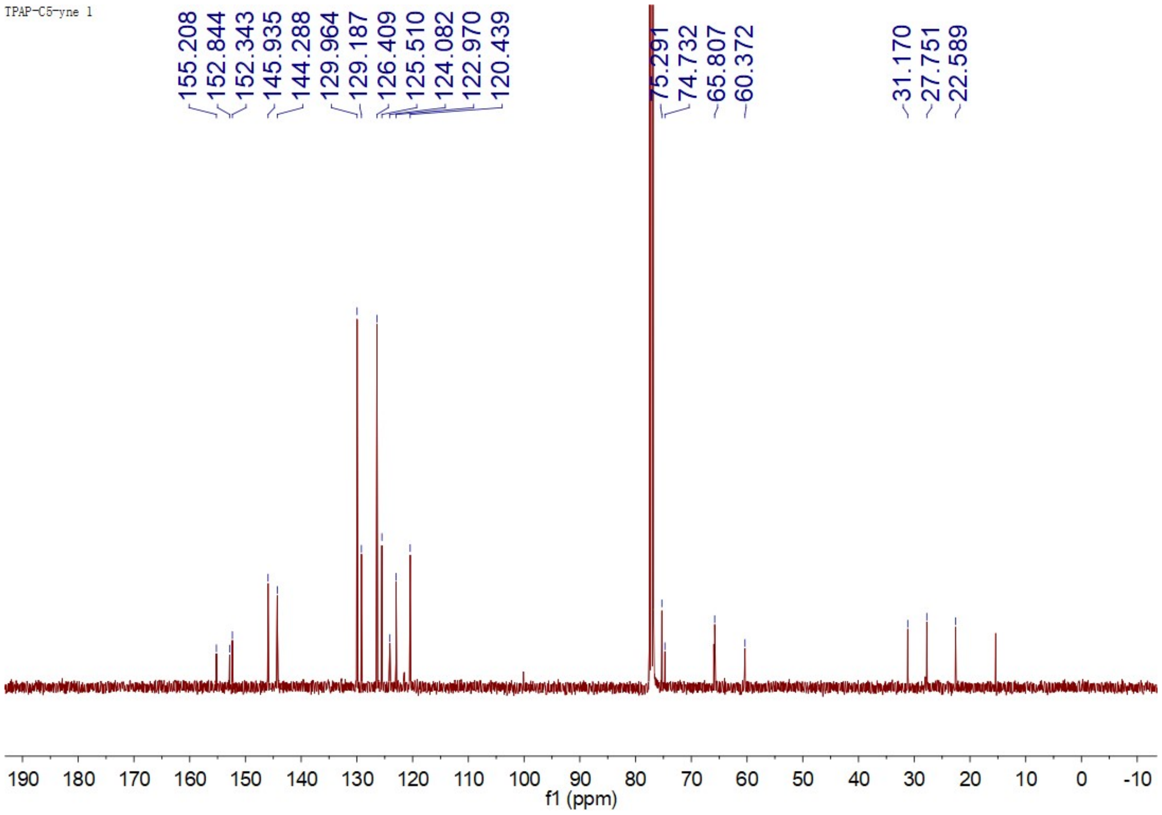

Figure S8. ^{13}C NMR (100 MHz, $CDCl_3$) spectrum of TPAP-C5-yne.

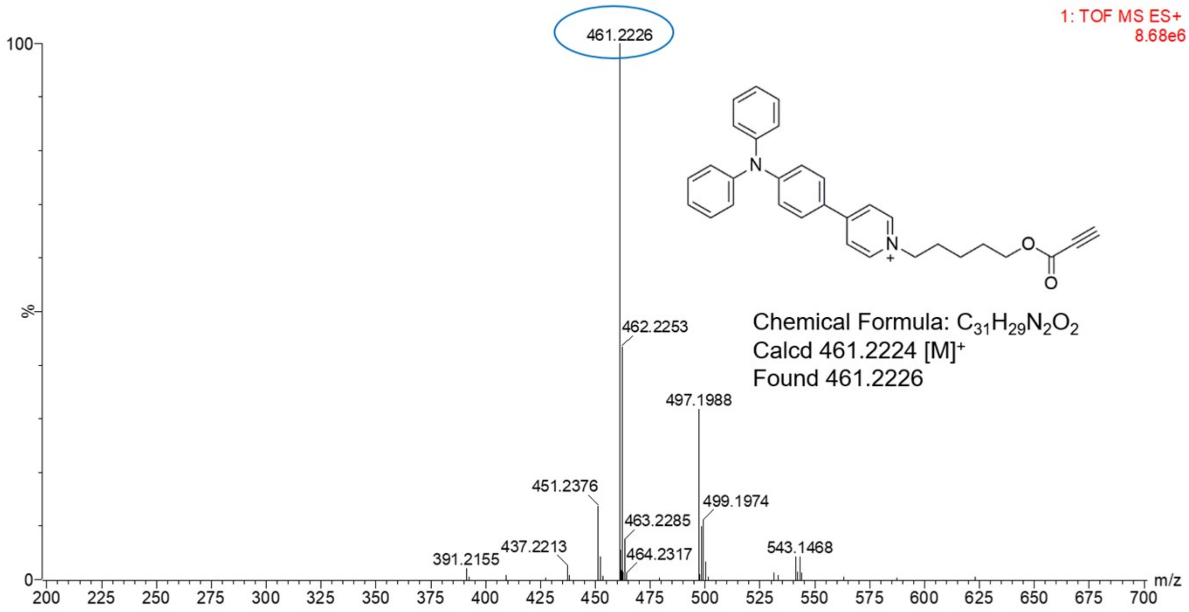

Figure S9. High-resolution mass spectrum of TPAP-C5-yne.

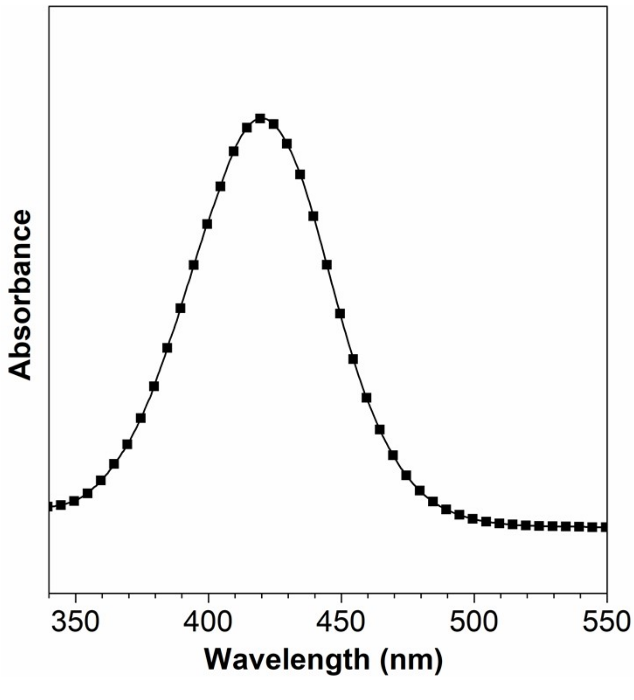

Figure S10. Absorption spectra of TPAP-C5-yne (10 μM) in THF.

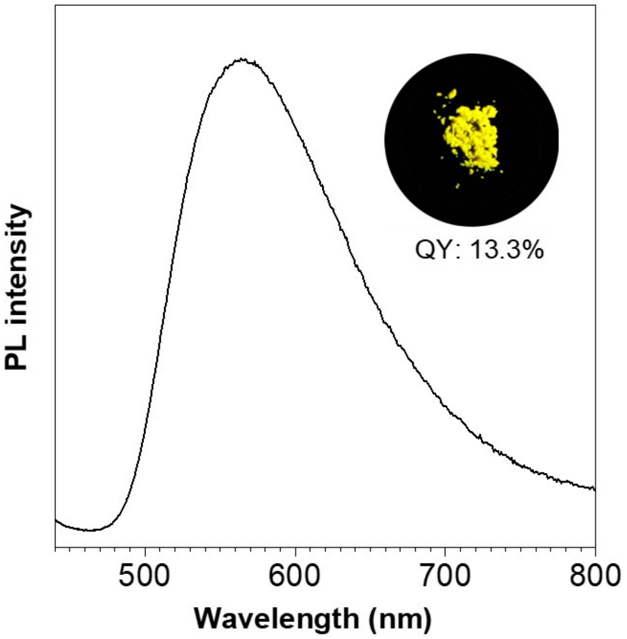

Figure S11. Fluorescence spectra of TPAP-C5-yne in solid state. Inset: photo of TPAP-C5-yne taken under a hand-hold UV lamp.

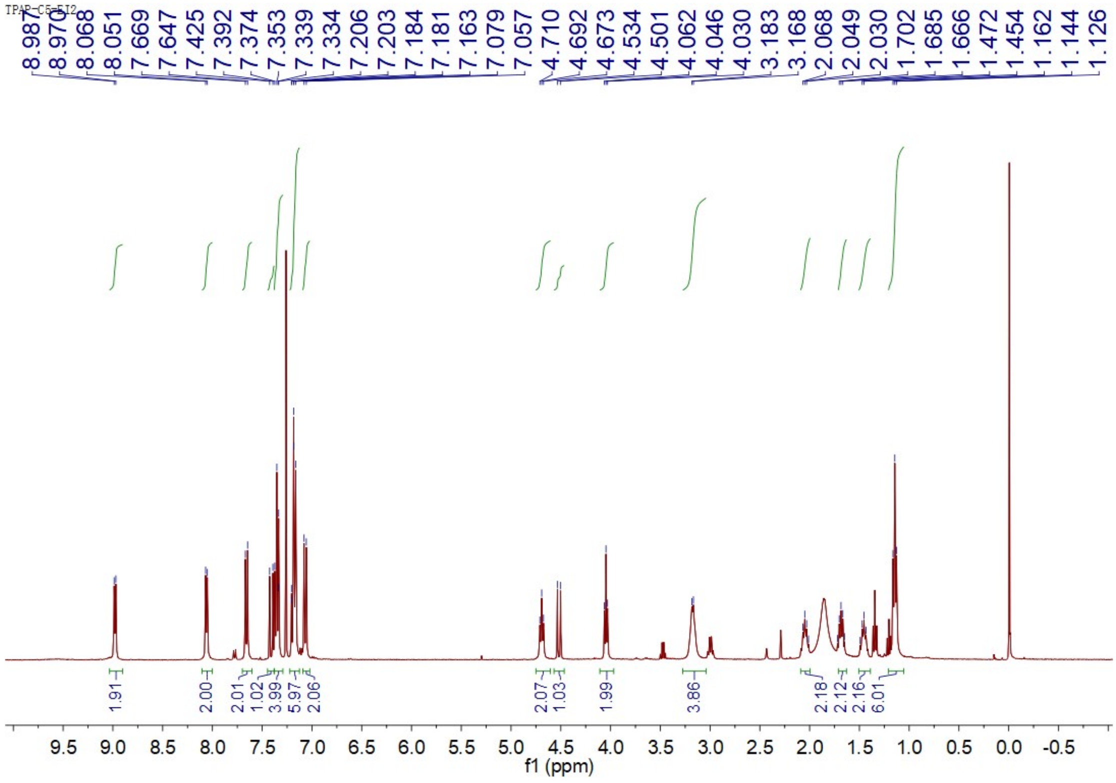

Figure S12. ^{1}H NMR (400 MHz, $CDCl_3$) spectrum of TPAP-C5-aa.

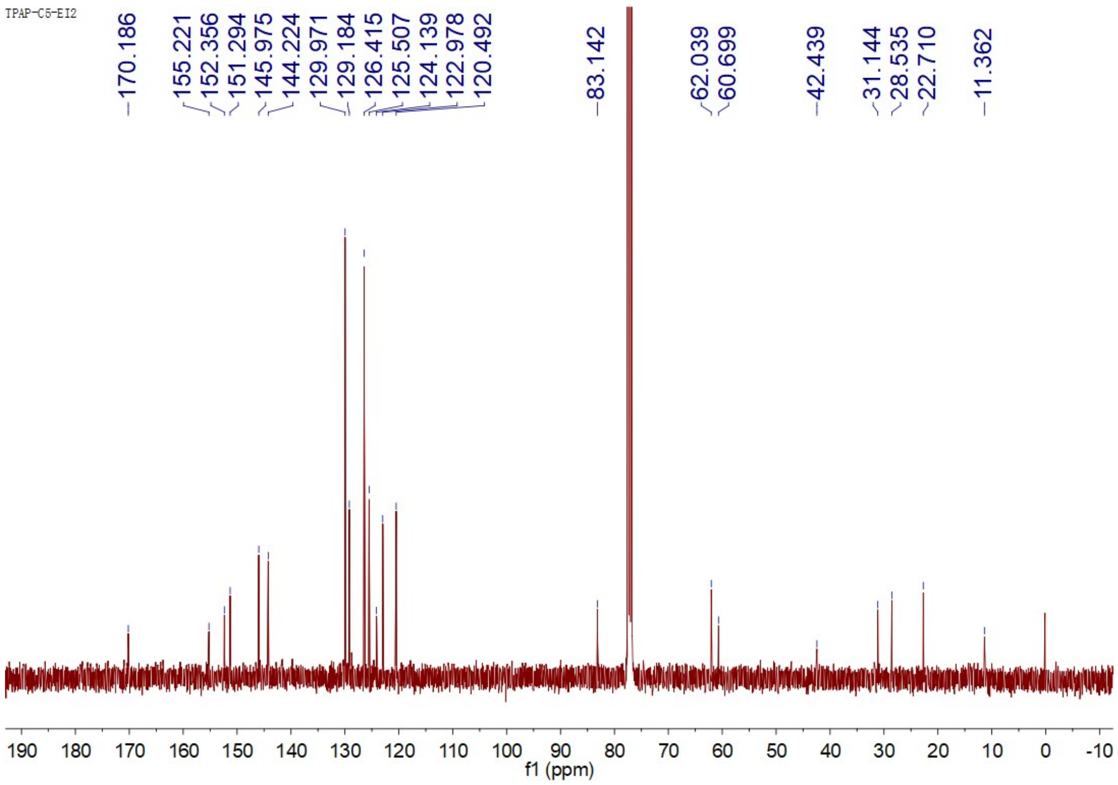

Figure S13. ^{13}C NMR (100 MHz, $CDCl_3$) spectrum of TPAP-C5-aa.

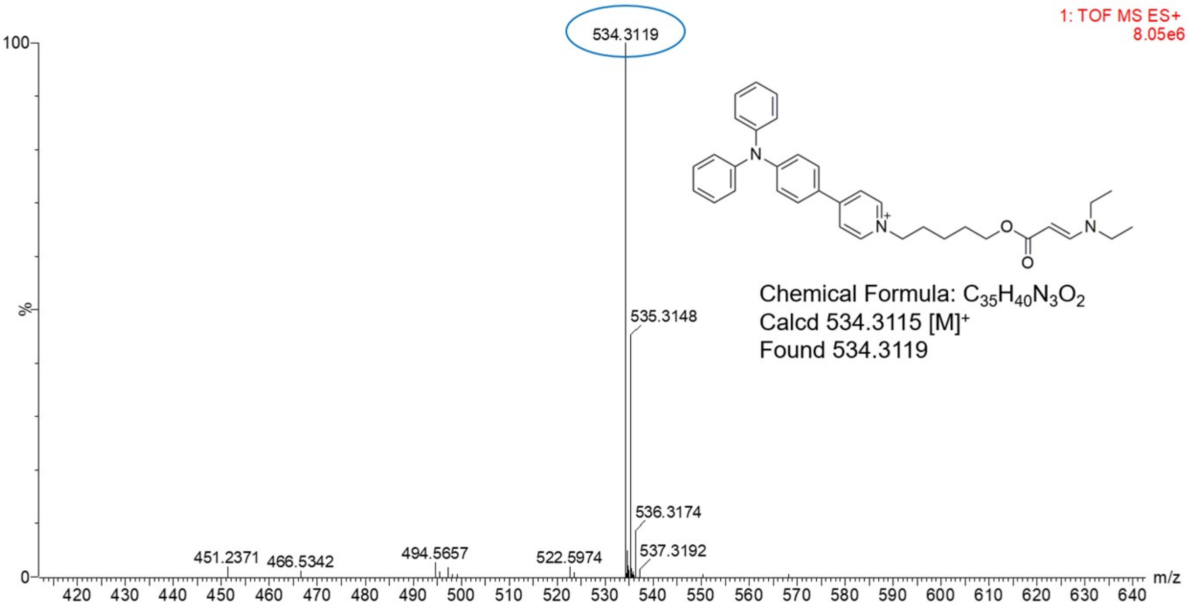

Figure S14. High-resolution mass spectrum of TPAP-C5-aa.

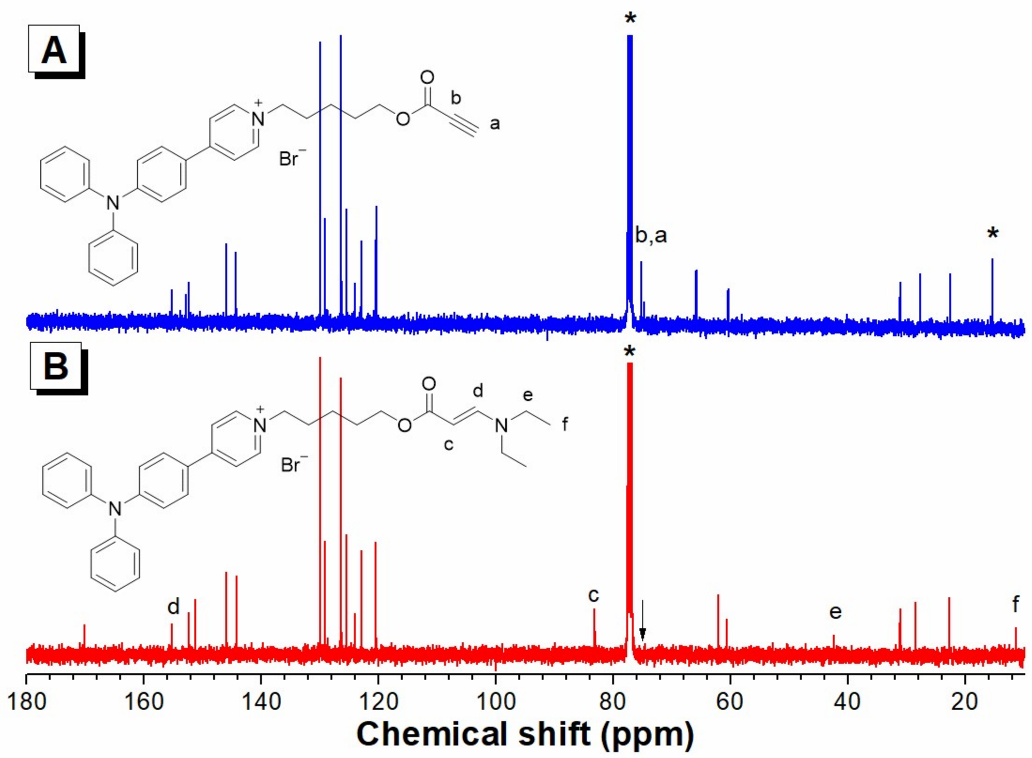

Figure S15. ^{13}C NMR spectra of (A) TPAP-C5-yne and (B) TPAP-C5-aa in $CDCl_3$. The solvent peaks are marked with asterisks.

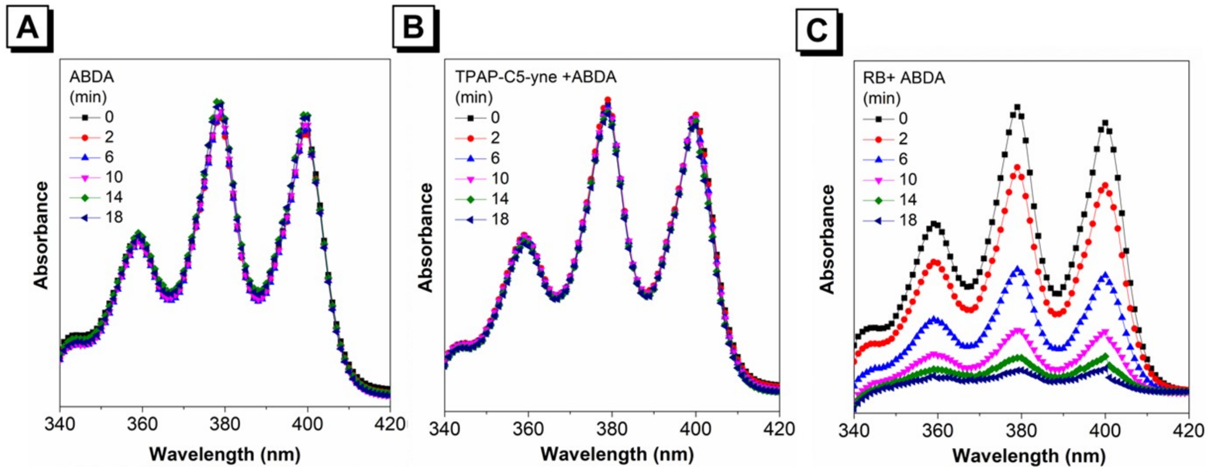

Figure S16. Time dependent absorption changes of ABDA (50 µM) mixed (a) without, (b) with TPAP-C5-yne (10 µM) and (c) with Rose Bangal (10 µM) upon visible light (λ = 400–700 nm, 4.76 mWcm^{-2}) irradiation.

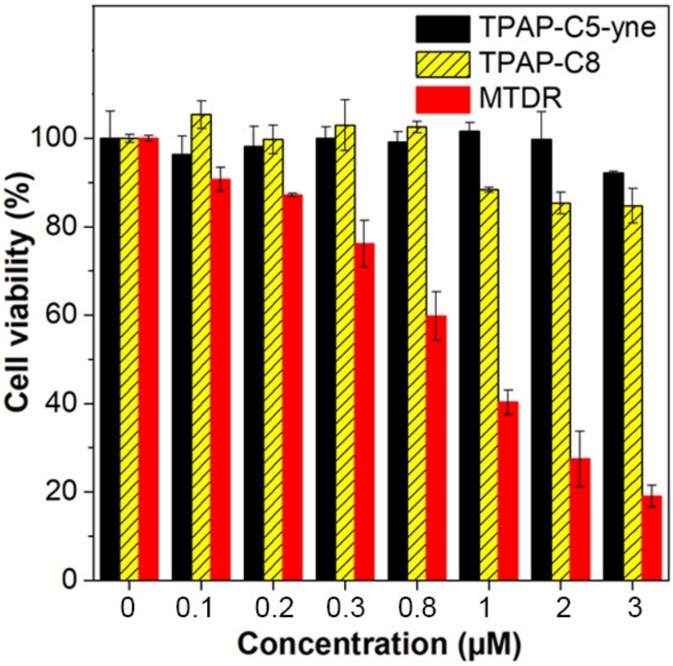

Figure S17. Cell viability of HeLa cells stained with different concentrations of TPAP-C5-yne, TPAP-C8 and MTDR for 24 h.

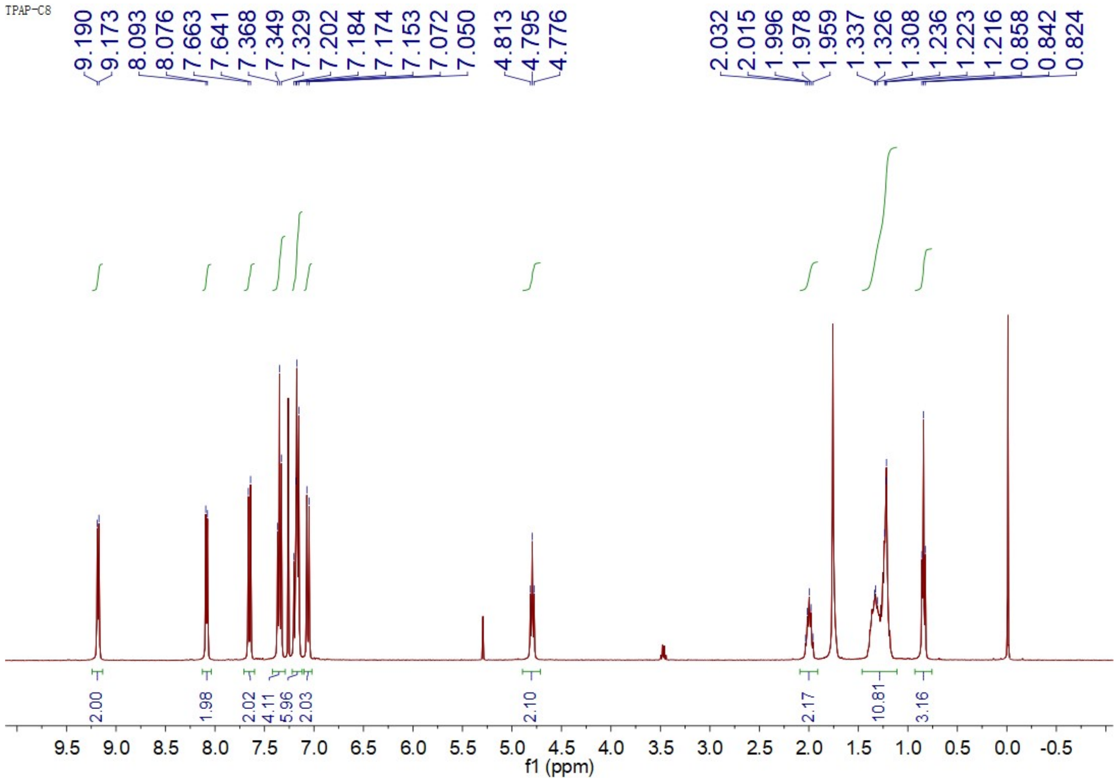

Figure S18. ^{1}H NMR (400 MHz, $CDCl_3$) spectrum of TPAP-C8.

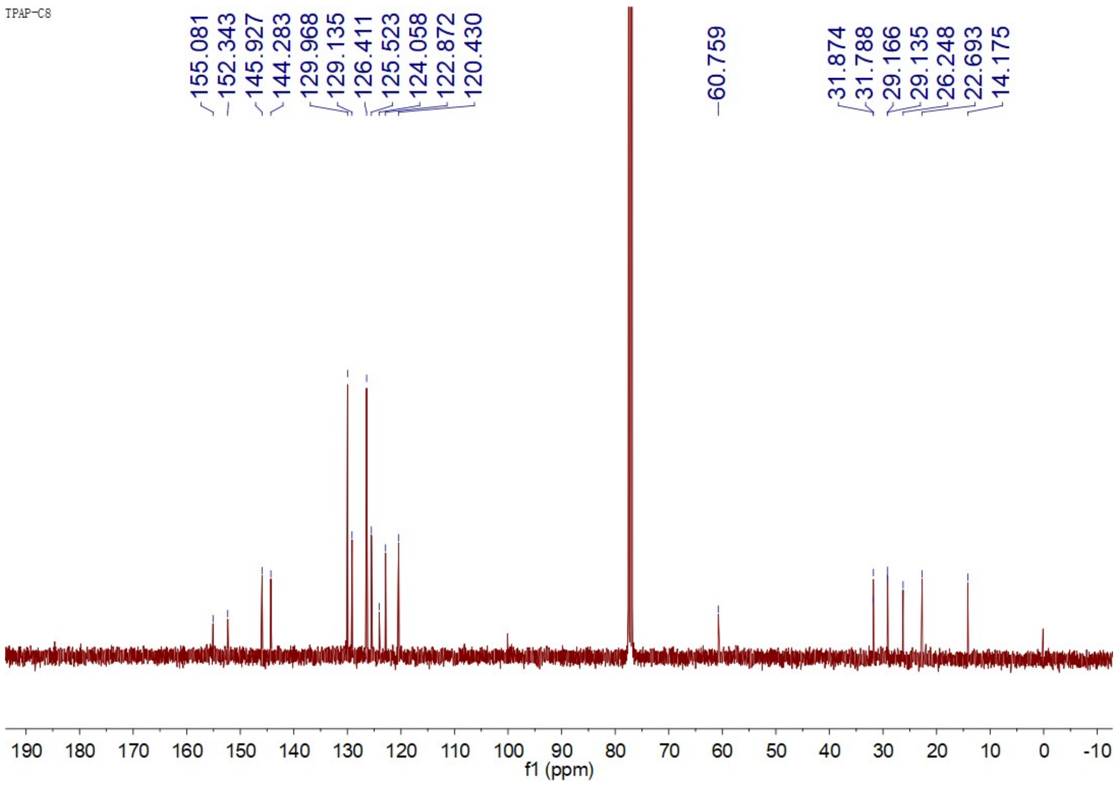

Figure S19. ^{13}C NMR (100 MHz, $CDCl_3$) spectrum of TPAP-C8.

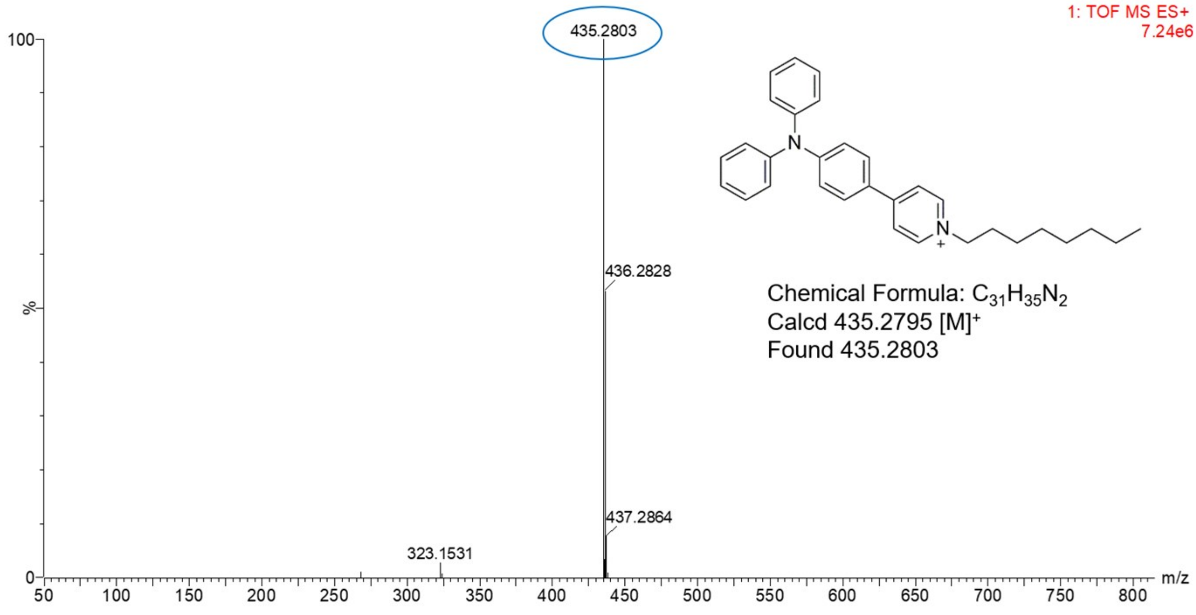

Figure S20. High-resolution mass spectrum of TPAP-C8.

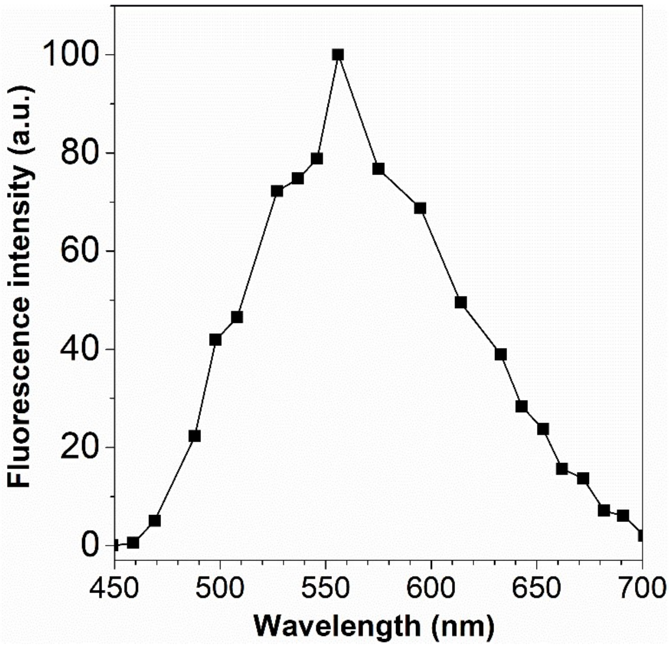

Figure S21. *In situ* fluorescence spectra of TPAP-C5-yne (300 nM) in HeLa cells.

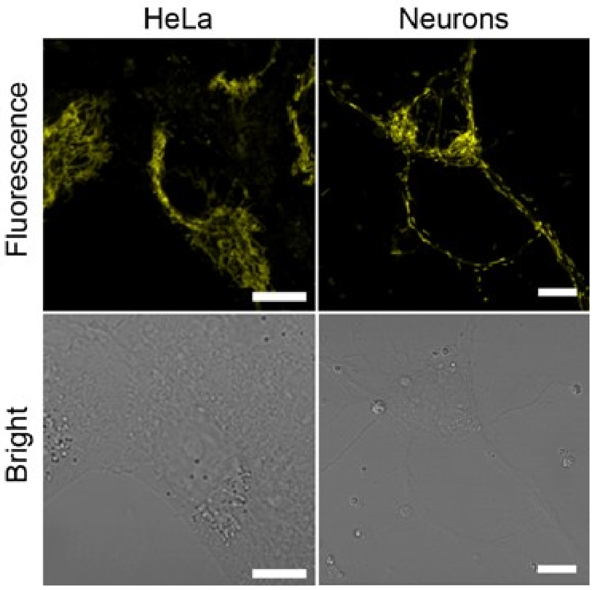

Figure S22. Confocal laser scanning microscopy images of live HeLa cells and primary rat hippocampal neurons stained with TPAP-C5-yne (300 nM). Scale bar: 10 μm.

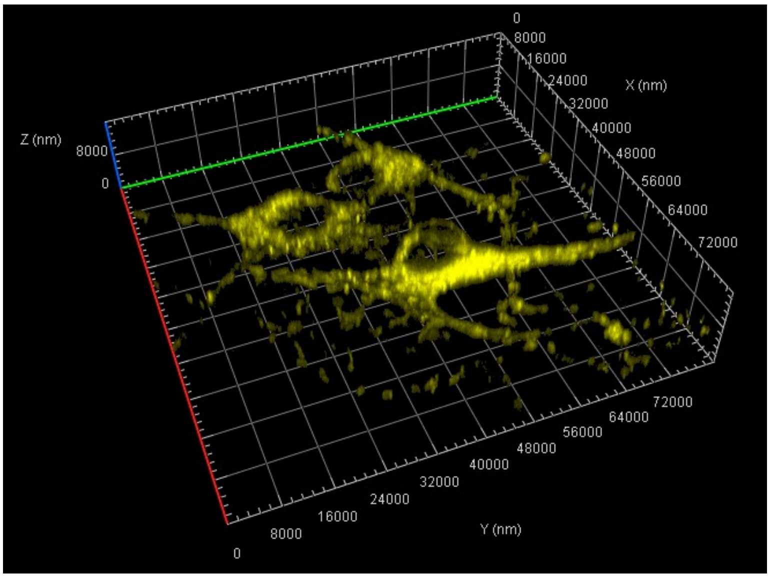

Figure S23. 3D confocal laser scanning microscopy images of rat hippocampal primary neurons stained with TPAP-C5-yne. Concentration: 300 nM.

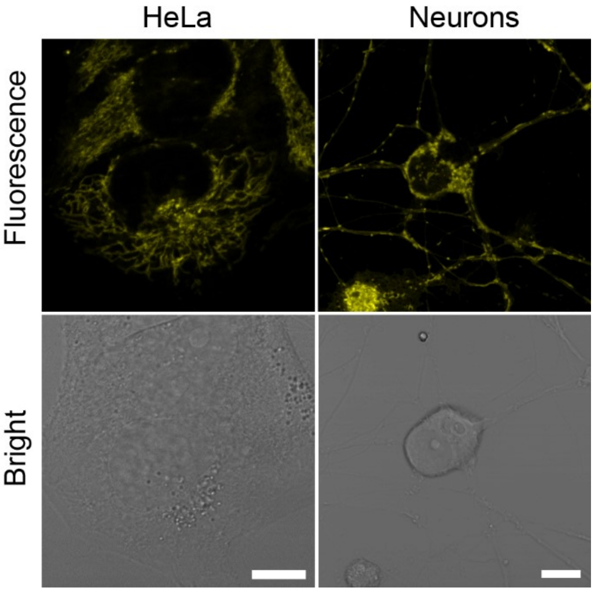

Figure S24. Confocal laser scanning microscopy images of live HeLa cells and primary rat hippocampal neurons stained with TPAP-C8 (300 nM). Scale bar: 10 µm.

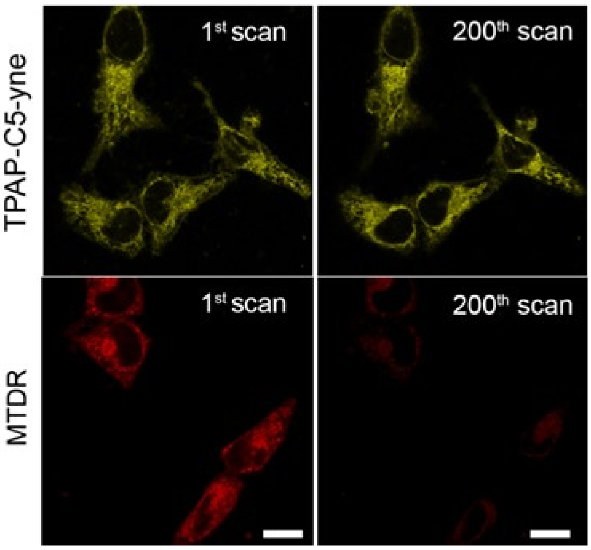

Figure S25. 1st and 200th confocal laser scanning microscopy images of live HeLa cells stained with TPAP-C5-yne (300 nM) and MTDR (300 nM).

References

[1] G. Liu, R. W. Tsien, *Neuropharmacology* **1995**, *34*, 1407.

[2] C. Yu, C. H. Li, S. Chen, H. Yoo, X. Qin, H. Park, *Sci. Rep.* **2018**, 8, 16976.

[3] H. Park, Y. Li, R.. Tsien, *Science* **2012**, *335*, 1362.

[4] A. Daoust, Y. Saoudi, J. Brocard, N. Collomb, C. Batandier, M. Bisbal, M. Salomé, A. Andrieux, S. Bohic, E. L. Barbier, *Bio. Protoc.* **2015**, 5, e1368.
